# Synthesis of p-Co_3_O_4_/n-TiO_2_ Nanoparticles for Overall Water Splitting under Visible Light Irradiation

**DOI:** 10.3390/nano6080138

**Published:** 2016-07-27

**Authors:** Qiang Zhang, Zhenyin Hai, Aoqun Jian, Hongyan Xu, Chenyang Xue, Shengbo Sang

**Affiliations:** 1 Key Laboratory of Instrumentation Science and Dynamic Measurement of Ministry of Education, North University of China, Taiyuan 030051, China; zhangq0902@163.com (Q.Z.); zhenyinhai0351@gmail.com (Z.H.); 2 MicroNano System Research Center, Key Laboratory of Advanced Transducers and Intelligent Control System of Ministry of Education and Shanxi Province & College of Information Engineering, Taiyuan University of Technology, Taiyuan 030024, China; jianaoqun@tyut.edu.cn; 3 School of Materials Science and Engineering, North University of China, Taiyuan 030051, China; xuhongyan@foxmail.com

**Keywords:** nanocomposites, photocatalysis, visible light, overall water splitting

## Abstract

p-Co_3_O_4_/n-TiO_2_ nanoparticles (~400 nm) for photocatalysis were prepared via carbon assisted method and sol-gel method in this work. The paper also studied the application of visible light illuminated p-Co_3_O_4_/n-TiO_2_ nanocomposites cocatalyst to the overall pure water splitting into H_2_ and O_2_. In addition, the H_2_ evolution rate of the p-Co_3_O_4_/n-TiO_2_ nanocomposites is 25% higher than that of the pure Co_3_O_4_ nanoparticles. Besides, according to the results of the characterizations, the scheme of visible light photocatalytic water splitting is proposed, the Co_3_O_4_ of the nanocomposites is excited by visible light, and the photo-generated electrons and holes existing on the conduction band of Co_3_O_4_ and valence band of TiO_2_ have endowed the photocatalytic evolution of H_2_ and O_2_ with higher efficiency. The optimal evolution rate of H_2_ and O_2_ is 8.16 μmol/h·g and 4.0 μmol/h·g, respectively.

## 1. Introduction

The sharp increase in global energy consumption makes efficient utilization of solar energy more urgent [1]. Therefore, overall water splitting under visible light has received much attention for production of renewable hydrogen from water [2]. To improve the efficiency of the hydrogen production, researchers work hard on modifying the nanomaterials [3,4,5,6]. Moreover, doping rare metals on semiconductor nanomaterials, changing the morphology of the nanomaterials and synthesis of complex nanomaterials are hot means that can be employed to improve the photocatalytic activity [7,8,9,10,11,12,13,14,15,16,17,18]. Maeda studied the photocatalytic activity of Rutile TiO_2_ doped by Ru, Rh, Ir, Pt or Au, with the results showing that the most water splitting amount of H_2_ and O_2_ for 4 h is 56.6 μmol and 26.5 μmol, respectively, when Pt doping amount is at 1 wt. % [7]. The water splitting amount of H_2_ for 8 h is 2750 μmol, which is photocatalyzed by 3D ZnO microspheres prepared by Guo [8]. Co_3_O_4_ Quantum Dots show excellent performance on photocatalytic water splitting that the water splitting amount of H_2_ and O_2_ is 0.79 μmol/h and 0.4 μmol/h, respectively [18]. Meanwhile, some nanomaterials (La_2_Ti_2_O_7_, PbTiO_3_, SrTiO_3_, etc.) also play important roles in water splitting [9,10,11,12,13,14,15,16,17]. The water splitting amount of H_2_ is 166.67 μmol/h·g when Au and reducing graphene oxide doped La_2_Ti_2_O_7_ act as photocatalyst [11]. However, the methods of synthesizing the photocatalyst mentioned above are complex, along with high cost of doping Pt. Therefore, this paper aims to design a photocatalyst with relatively high activity via easy methods and low cost.

Many researches focus on enhancing the visible light absorption of TiO_2_, but the main problems of these methods are high cost, complex processes and compositions [19,20,21,22]. Band gap of the Co_3_O_4_ nanomaterials is very close to the wavelength of visible light, so Co_3_O_4_ nanomaterials can be excited by visible light. However, typically, the p type Co_3_O_4_ semiconductor cannot be used on overall water splitting [23]. Some studies researched on changing the band gap of Co_3_O_4_; nevertheless, the constraints of these methods are high cost and difficult controlling processes [12,13,14,15,16,17,24]. Therefore, a new p-Co_3_O_4_/n-TiO_2_ photococatalyst is designed for overall pure water splitting under visible light irradiation, and the Co_3_O_4_ and TiO_2_ nanoparticles is, to the best of our knowledge, combined in nano-scale for the first time [25,26,27]. According to the band edge positions of TiO_2_-Co_3_O_4_ nanocomposites, the excited electrons on the conduction band of the p-type Co_3_O_4_ transfer to that of the n-type TiO_2_, and simultaneously holes on the valence band of n-TiO_2_ can be transferred to that of p-Co_3_O_4_ under the potential of the band energy difference. The migration of photogenerated carriers can be promoted by the internal field, which results in existence fewer barriers. Therefore, the electron–hole pairs recombination of can be reduced, and the photocatalytic reaction can be improved greatly [28,29].

Many efforts have been devoted to synthesizing Co_3_O_4_ with well-controlled dimensionality, sizes, and crystal structure [30,31,32,33]. Wang et al. reported a carbon-assisted carbothermal method to synthesize the single-crystalline Co_3_O_4_ octahedral cages with tunable surface aperture [30]. Moreover, with the carbon spheres obtained through hydrothermal carbonization as the sacrificial template, Du et al. have successfully synthesized Co_3_O_4_ hollow spheres by a one-pot calcinations method [31]. Furthermore, Zhang et al. described the synthesis of high purity octahedral Co_3_O_4_ with the help of carbon materials using one-step microwave reaction [33]. Based on the researches mentioned above, p-Co_3_O_4_ nanoparticles are prepared through a more facile and environment-friendly carbon-assisted method using degrease cotton, which have been reported by our group in 2015 [23]. TiO_2_ nanoparticles were composed via gol-sel method [34]. Besides, the paper presented the study on the application of visible light illuminated p-Co_3_O_4_/n-TiO_2_ nanocomposites cocatalyst to the overall pure water splitting into H_2_ and O_2_, and the H_2_ evolution rate of the p-Co_3_O_4_/n-TiO_2_ nanocomposites is 25% higher than that of the pure Co_3_O_4_ nanoparticles. In addition, the scheme of visible light photocatalytic water splitting is proposed based on the results of the characterizations.

## 2. Experimental Section

### 2.1. Synthesis

All chemicals were reagent grade and used without further purification. Cobalt nitrate, tetra-butyl ortho-titanate (TBOT), ethanol, hydrochloric acid and nitric acid were purchased from Sinopharm Chemical Reagent Co. Ltd. (Shanghai, China). Commercial degreasing cotton (Pagoda Medical Devices Co., Ltd., Dingzhou, China) was used as the reactant. Deionized water of 18.25 MΩ was purified through an ultra-pure (UPR) system (Xi'an You Pu Equipment Co., Ltd., Xi'an, China).

The Co_3_O_4_ nanoparticles (0.06 mol) were prepared via environmentally friendly carbon-assisted method, as reported in the previous article [23]. Specifically, immersed into 20 mL Co(NO_3_)_2_ pink solution (3 mol/L), 1.5 g degreasing cotton was then kept in an ultrasonic bath for 10 min in order to get a good dispersion of Co^2+^ on the surface of degreasing cotton. Then, the treated degreasing cotton was collected and transferred into a quartz petri dish in the tube furnace (OTF-1200X-III, Hefei Ke Jing Materials Technology Co., Ltd., Hefei, China) and kept at 600 °C for 2 h in the air. Besides, the Co_3_O_4_ powders were cooled to the room temperature naturally.

The Co_3_O_4_ nanoparticles were composed with 0.06 mol TiO_2_ through the following steps: The Co_3_O_4_ powders were put into a beaker with solation whose volume ratio of TBOT, nitric acid, alcohol and deionized water were 30.3:0.6:12.5:1 with continuous stirring. Moreover, a multifunctional magnetic stirrer (MPL-CJ-88) was used to stir and heat the solution (Jintandadi Automation Factory, Jintan, China). With the help of ultrasonic bath (KQ-2500DE) from Kunshan Shumei Ultrasonic Instrument Co. Ltd. (Kunshan, China), the beaker of solution and 10 g of particles (the obtained TiO_2_ solution is about 150 mL) were ultra-sonicated, after which it was stirred for 30 min. The wet particles were heated at 500 °C for 2 h after being reacted for three hours. After milling with an agate mortar, the TiO_2_-Co_3_O_4_ composite was finally obtained.

### 2.2. Characterization

The crystal structure of the sample was measured through X-ray diffraction (XRD, a Bruker D8, λ = 1.5406 Å) (Bruker (Beijing) Technology Co., Ltd., Beijing, China) in the 2θ of 10°–80° with a scan rate of 10°/min and Cu Kα radiation, at 40 KV. Chemical composition analysis was carried out using X-ray photoelectron spectroscopy (XPS). This XPS was collected using an ESCALAB 250Xi spectrometer (Shanghai Hu Yueming scientific instruments Ltd., Shanghai, China) with a standard Al Kα radiation which was provided with the binding energies calibrated based on the contaminant carbon (C1s = 284.6 eV). The morphology was observed by transmission electron microscopy (TEM, JEOL JEM-2011) (Guangzhou Office of Japan Electronics Co., Ltd., Guangzhou, China). Furthermore, with the presence of BET (Brunauer-Emmet-Teller) and BJH (Barrett-Joyner-Halenda), specific surface areas and pore size distributions were computed from the results of N_2_ physisorption at 77 K (Micromeritics ASAP 2020) (Micromerics (Shanghai) Instrument Co., Ltd., Shanghai, China). A Cary 300 Scan Ultraviolet–visible (UV-Vis) spectrophotometer (Shanghai Precision Instrument Science Co., Ltd., Shanghai, China) was employed to record the UV-Vis diffuse reflectance spectra (DRS) in a region of 200 to 800 nm.

### 2.3. Photocatalysis

**Figure 1:**
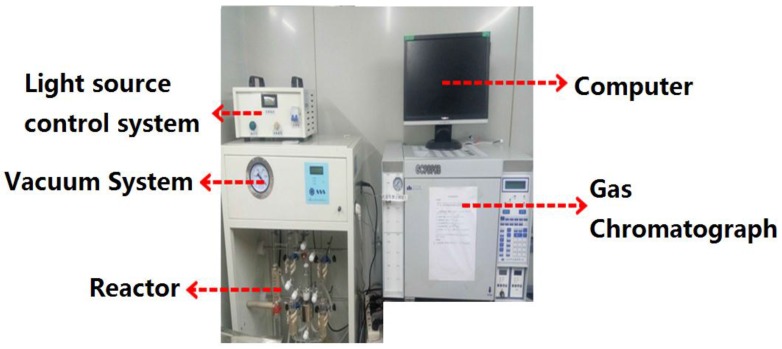
Diagram of visible light water splitting system.

The photocatalytic activity for the overall pure water splitting into H_2_ and O_2_ was estimated under visible light condition. The diagram of visible light water splitting system is shown in Figure 1.

Typically, 0.02 g photocatalyst was added into the solution (~200 mL) containing 200 mL pure water solution. After put in an ultrasonic stirring for 20 min, purged by Ar gas for 20 min, the mixture was then irradiated under visible light with magnetic stirring. A 300 W Xe arc lamp (LSH-A500, KaifengHxsei Science Instrument Co. Ltd., Kaifeng, China) with UV cut-off filters (420 nm) was used as the light source. In addition, the hydrogen produced was analyzed by a gas chromatography (GC-9890B, Shanghai Linghua Instrument Co. Ltd., Shanghai, China) equipped with a thermal conductivity detector and a stainless steel column packed with molecular sieve (5 A). Ar gas (99.999%) was used as the carrier gas.

## 3. Results and Discussion

**Figure 2:**
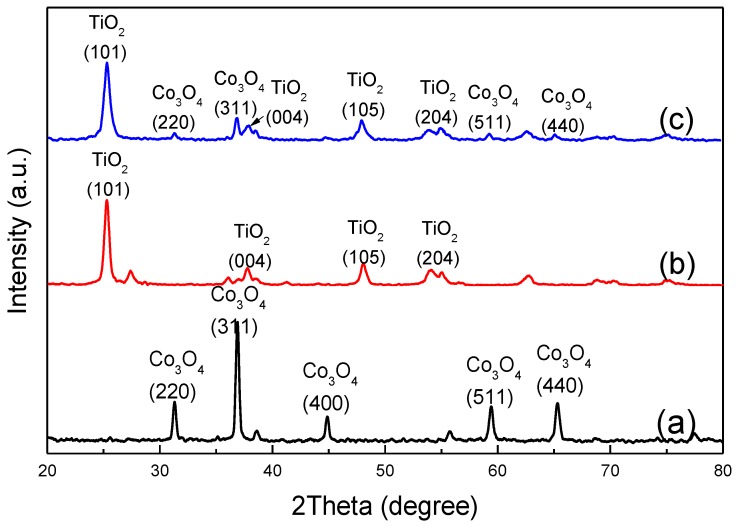
X-ray diffraction (XRD) pattern of the: (**a**) Co_3_O_4_ sample; (**b**) TiO_2_ sample; and (**c**) TiO_2_-Co_3_O_4_ sample. a.u.: any unit.

The XRD patterns of the Co_3_O_4_ are shown in Figure 2a. All peaks have a good agreement with the standard spinel cubic Co_3_O_4_ spectrum (JCPDS No. 42-1467), while there are no impurity peaks found in the XRD patterns. The result suggests that the well-crystallized Co_3_O_4_ with high purity sample is produced. According to Scherrer’s formula, *D* = 0.89λ/(*B*cosθ) (where *D* is the average dimension of crystallites; λ is the X-ray wavelength; θ is the Bragg Angle; and *B* is the pure diffraction broadening of a peak at half-height, which is calculated according to the data of XRD spectrum), the crystalline size of Co_3_O_4_, calculated from the strongest peak, locating at (311) plane, are estimated to be 51.64 nm.

Figure 2b demonstrates that anatase (JCPDS No. 21-1272) with high purity sample is obtained via gol-sel method. According to Scherrer’s formula mentioned above, the crystalline size of TiO_2_ is estimated to be 22.48 nm. The wider peaks in Figure 2c have shown bigger composites sizes. To confirm all of the nanoparticles contain both Co_3_O_4_ and TiO_2_, the TiO_2_ are compounded after the obtaining of bigger size Co_3_O_4_. In addition, avoiding the visible light absorber Co_3_O_4_ being coated completely, the mol ratio of these two materials is controlled strictly as 1:1. As demonstrated in Figure 2c, there are no facets existing other than Co_3_O_4_ and TiO_2_. However, the Co_3_O_4_ peaks are much weaker than that of TiO_2_, with the possible reason being that the Co_3_O_4_ is partially coated by TiO_2_.

**Figure 3:**
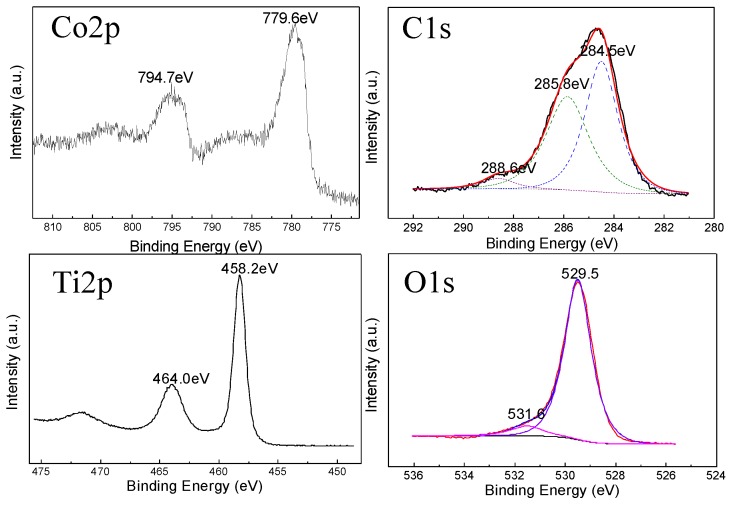
X-ray photoelectron spectroscopy (XPS) spectra of the TiO_2_-Co_3_O_4_ sample.

To identify the chemical state of the nanocomposite, the X-ray photoelectron spectroscopy (XPS) was measured (Figure 3). By using adventitious carbon at 284.8 eV, the XPS spectra were corrected for sample charging. The Co2p orbital showed splitting peaks at 794.7 and 779.6 eV, representing Co2p 3/2 and Co2p 1/2 [35]. The Ti2p orbital showed peaks at 464.0 and 458.2 eV, thus indicating the Ti2p 3/2 and Ti2p 1/2 [36,37]. As for the XPS of O1s at 531.6 and 529.5 eV, it would indicate the presence of adsorbed water and oxygen in the near-surface region [13]. The strong peak of C1S centers at 284.5 eV can be assigned to elemental carbon, which has given rise to the incomplete burning of degreasing cotton; in contrast, the other two peaks appeared at 285.8 and 288.6 eV, respectively, which are ascribed to the O=C–O bonds and (CHO)*_x_* from insufficient combustion residual degreasing cotton [38]. No Co–C or Ti–C band is found in the spectra, which means that the conjunctions of the nanocomposites are not affected by C. Therefore, according to the results of the XPS spectra, the TiO_2_-Co_3_O_4_ sample is composed only by TiO_2_, Co_3_O_4_ and residual degreasing cotton, which is consisted with the XRD results.

**Figure 4:**
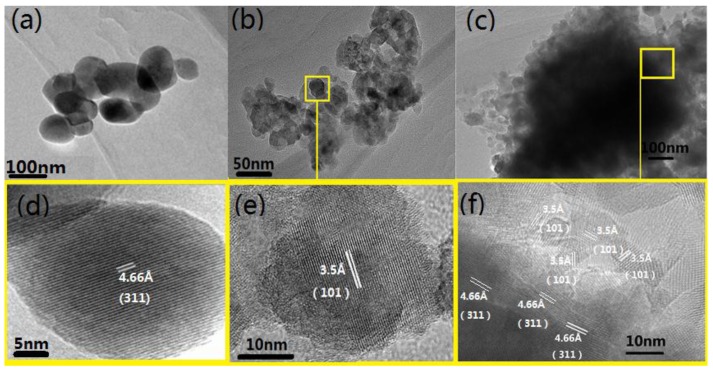
Transmission electron microscopy (TEM) images of: (**a**) the Co_3_O_4_ sample; (**b**) TiO_2_ sample; and (**c**) TiO_2_-Co_3_O_4_ sample. High Resolution Transmission Electron Microscopy (HRTEM) images of: (**d**) the Co_3_O_4_ sample; (**e**) TiO_2_ sample; and (**f**) TiO_2_-Co_3_O_4_ sample.

The morphology and structure of the samples are observed via TEM, with the results presented in Figure 4. Figure 4a shows that the Co_3_O_4_ nanospheres with a relatively uniform size of 60 nm are obtained. It can be seen that 20 nm TiO_2_ nanoparticles compose the big group in Figure 4b, which is consistent with the calculation from XRD mentioned above. The Figure 4c is the TEM image of the TiO_2_-Co_3_O_4_ sample. The darker part in Figure 3c is Co_3_O_4_ while the lighter part is TiO_2_, which can be proved by both the synthetic procedures and the results of High Resolution Transmission Electron Microscopy (HRTEM) in Figure 4d–f. The TiO_2_ accumulates around the Co_3_O_4_ incompletely, while the TiO_2_ and the Co_3_O_4_ connected closely at the interface of the composite. The result of Figure 2, Figure 3 and Figure 4 improves the elements and framework of the p-Co_3_O_4_/n-TiO_2_ nanocomposites.

**Figure 5:**
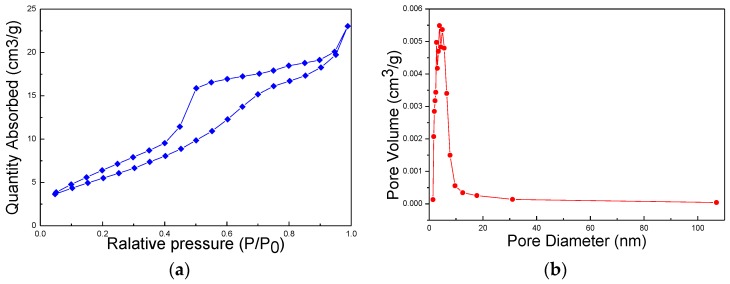
(**a**) Nitrogen adsorption–desorption isotherms; and (**b**) Barrett-Joyner-Halenda (BJH) pore size distributions of TiO_2_-Co_3_O_4_ sample.

The Co_3_O_4_ is prepared firstly due to its clear edge and bigger size, while the mesoporous structure of the TiO_2_ (which is shown as Figure 5) is another reason for the preparation of its designing on step two. Larger area of the conjunction and larger surface area of the composite for higher photocatalytic efficiency can be obtained in this way. Brunauer-Emmett-Teller (BET) surface areas and Barrett-Joyner-Halenda (BJH) pore size distributions of the TiO_2_-Co_3_O_4_ are shown in Figure 5. According to the International Union of Pure and Applied Chemistry (IUPAC) classification, the isotherms exhibit type IV. As for the increase in the uptake of N_2_ at intermediate pressure, which suggests the existence of mesoporous resulted from the interparticle space in the samples, it can facilitate the water accessibility to nanoparticles. According to the corresponding BJH pore size distribution curve, the pore size distribution has a relatively intense peak at ~10 nm. The BET surface area is calculated to be 39.64 m^2^/g and the average pore size is 3.83 nm. These results and the results of the TEM demonstrate the mesoporous existence on the TiO_2_ layer of the composites. Furthermore, the relative higher surface area and the mesoporous structure will play a very important role in improving the water splitting efficiency of the composite.

**Figure 6:**
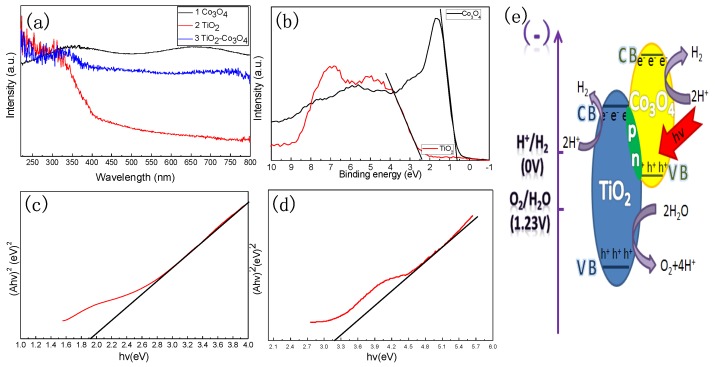
(**a**) Ultraviolet–visible (UV-Vis) spectra; and (**b**) Valence-band XPS spectra of TiO_2_ and Co_3_O_4_ nanoparticles. (Ahv)^2^–hv curve of: (**c**) Co_3_O_4_ nanoparticles; and (**d**) TiO_2_ nanoparticles. (**e**) Schematic diagram of the water splitting reaction of the TiO_2_-Co_3_O_4_ heterostructures.

Investigation was conducted on the optical absorption properties of the TiO_2_ nanoparticles, Co_3_O_4_ nanoparticles and TiO_2_-Co_3_O_4_ heterostructures at room temperature by UV-Vis spectroscopy, as shown in Figure 6a. Broad background absorption in the visible light region can be observed for Co_3_O_4_ (Figure 6a, curve 1), while there is no absorption invisible light region for TiO_2_ (Figure 6a curve 2). However, the absorption of the TiO_2_-Co_3_O_4_ composite in the visible light region can achieve great improvement owing to the Co_3_O_4_ (Figure 6a curve 3). This result illustrates that the TiO_2_-Co_3_O_4_ composite can be irradiated by visible light. Importantly, Figure 6b shows the valence-band XPS spectra of TiO_2_ and Co_3_O_4_, clearly indicating that the valence band maximum of TiO_2_ and Co_3_O_4_ are 2.4 and 0.6 eV. In addition, according to that the UV-Vis spectroscopy of Co_3_O_4_, the bandgap value calculated is 1.9 eV (Figure 6c) while the bandgap Eg value of TiO_2_ is 3.2 eV (Figure 6d). Based on the above results, the schematic diagram of the water splitting reaction of the TiO_2_-Co_3_O_4_ heterostructures is shown as Figure 6e. When Co_3_O_4_ semiconductor absorbs visible light photons, electrons in the valence band are excited to the conduction band. As a result, excited electrons and holes are generated in the conduction and valence bands of the composite, respectively. These photogenerated carriers drive reduction and oxidation reactions. The reduction of water to hydrogen and oxidation of reduced redox mediators occurs on Co_3_O_4_ and TiO_2_ concurrently with the reduction of oxidized redox mediators and oxidation of water to oxygen on the TiO_2_ [2]. According to the band edge position, the excited electrons on the conduction band of the p-type Co_3_O_4_ transfer to that of the n-type TiO_2_, and simultaneous holes on the valence band of n-TiO_2_ can be transferred to that of p-Co_3_O_4_ under the potential of the band energy difference. The migration of photogenerated carriers can be promoted by the internal field, so barely any barrier exists. Therefore, the electron–hole pairs recombination can be reduced, and the p-n junction has a significant impact on the efficiency of photocatalytic water splitting.

**Figure 7:**
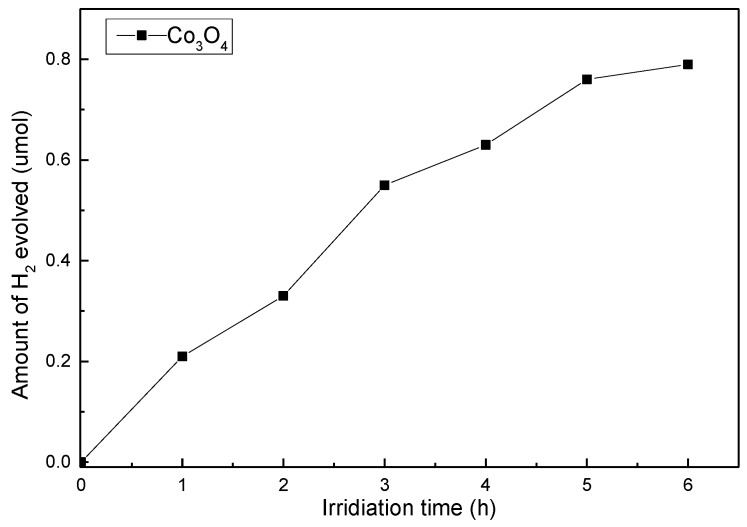
Photocatalytic H_2_ evolution on Co_3_O_4_ nanocomposites under visible-light irradiation using 0.02 g photocatalyst suspended in 200 mL pure water solution in a Pyrex glass cell.

The results of the measurements of H_2_ evolution through direct photocatalytic water splitting with Co_3_O_4_ nanocomposites under visible light are shown as Figure 7. The H_2_ evolution rates are 6.5 μmol/h·g, as shown in Figure 7.

**Figure 8:**
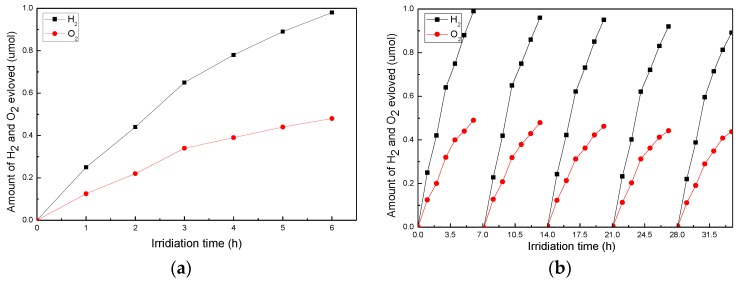
(**a**) Photocatalytic H_2_ and O_2_ evolution on Co_3_O_4_-TiO_2_ nanocomposites under visible-light irradiation using 0.02 g photocatalyst suspended in 200 mL pure water solution in a Pyrex glass cell; and (**b**) cycling measurements of H_2_ and O_2_ evolution through direct photocatalytic water splitting with Co_3_O_4_-TiO_2_ nanocomposites under visible light.

The measurements results of the H_2_ and O_2_ evolution through direct photocatalytic water splitting with Co_3_O_4_-TiO_2_ nanocomposites under visible light are shown in Figure 8. The H_2_ and O_2_ evolution rates are 8.16 μmol/h·g and 4.0 μmol/h·g, respectively, as shown in Figure 8a. The approximated 2:1 of the H_2_ and O_2_ generation ratio demonstrate the Co_3_O_4_-TiO_2_ nanocomposites capability for the overall water splitting. Due to the photoreduction of O_2_ via a reverse reaction during water splitting, the slight deviation of the H_2_:O_2_ ratio from the ideal stoichiometric value could thus be acquired. The hydrogen peroxide was formed as an oxidation product, and molecular oxygen (another oxidation product) adsorbed too intimately on the surface of photocatalyst so as to desorb the gas phase, thus resulting in the lack of O_2_ [7,19]. Through the comparison between Figure 7 and Figure 8a, it can be seen that the H_2_ evolution rate of the p-Co_3_O_4_/n-TiO_2_ nanocomposites is 25% higher than that of the pure Co_3_O_4_ nanoparticles. This result illustrates the mechanism in the way that the electron–hole pairs recombination can be reduced due to the structure of the p-Co_3_O_4_/n-TiO_2_ nanocomposites, while the p-n junction has a significant impact on the efficiency of photocatalytic water splitting. Figure 8b shows that the H_2_ and O_2_ evolution rates are 7.67 μmol/h·g and 3.92 μmol/h·g after being recycled five times. The slight reduction of the gas generation is resulted by the quality loss caused by powders cleaning and drying; additionally, the mesoporous structure of the nanomaterials makes the water molecules difficult to be removed thoroughly. Together, the capability of the Co_3_O_4_-TiO_2_ nanocomposites for the overall water splitting is demonstrated by the measurements of H_2_ and O_2_ evolution under visible light.

The gas generation rate comparison result is not precise because the light source and the sacrificial agent are different according to different researches. Therefore, a comparison was made of our results with some of the other works on pure water splitting under visible light. As concluded in Table 1, the gas generation rate of our nanocomposites is in the medium level. However, the preparation method of Co_3_O_4_ Quantum Dot [12] and Pt-TiO_2_ [7] are hard to control, and, needless to say, high cost. Besides, the materials of p-LaFeO_3_/Fe_2_O_3_ [39] are uncommon, and the treating temperature of Co-TiO_2_ preparation is extremely high (~1100 °C) [19]. The simple preparation method of p-Co_3_O_4_/n-TiO_2_ nanocomposites used in this work is environmentally friendly. In conclusion, the p-Co_3_O_4_/n-TiO_2_ nanocomposites presented in this paper has a relatively high photocatalytic activity in terms of its facilitative methods.

## 4. Conclusions

In this work, carbon assisted method and sol-gel method were used to obtain the nanocomposite p-Co_3_O_4_/n-TiO_2_ photocatalyst. Based on the XRD and XPS results, no other phase is generated other than Co_3_O_4_ and TiO_2_. The formation of the p-Co_3_O_4_/n-TiO_2_ conjunction is proven by the TEM and HRTEM investigations of Co_3_O_4_, TiO_2_ and Co_3_O_4_-TiO_2_ nanoparticles. The BET surface area and the average pore size of themes porous structure nanocomposite are 39.64 m^2^/g and 3.83 nm, respectively, which can be concluded through the Nitrogen adsorption-desorption curve. The schematic of visible light photocatalytic water splitting is surmised through the results of UV-Vis spectra, (Ahv)^2^–hv curve and valence-band XPS spectra of TiO_2_ and Co_3_O_4_ nanoparticles. The p-Co_3_O_4_/n-TiO_2_ composites have a significant impact on the efficiency of photocatalytic water splitting. Finally, the results of visible light irradiating overall water splitting reaction can prove the photocatalytic activity of the p-Co_3_O_4_/n-TiO_2_ nanocomposite. The optimal evolution rate of H_2_ and O_2_ is 8.16 μmol/g·L and 4.0 μmol/g·L, respectively. In addition, the H_2_ evolution rate of the p-Co_3_O_4_/n-TiO_2_ nanocomposites is 25% higher than that of the pure Co_3_O_4_ nanoparticles.

## Figures and Tables

**Table 1:** Research status of photocatalytic water splitting.

Reference	Materials	Light Source	H_2_ Amount	O_2_ Amount	Sacrificial Agent
[8]	3D ZnO microspheres	Visible light	343.75 μmol/h		Methanol, ethanol, formaldehyde
[12]	Co_3_O_4_ Quantum Dot	Visible light	0.79 μmol/h	0.4 μmol/h	none
[7]	Pt-TiO_2_	Ultraviolet light	56.6 μmol/h	26.5 μmol/h	none
[11]	Au@Pt-NLTO/rGO	Visible light	166.67 μmol/h·g		ethanol
[39]	p-LaFeO_3_/Fe_2_O_3_	Visible light	80 μmol/h	40 μmol/h	none
[19]	Co-TiO_2_	Visible light	11 μmol/h·g	5 μmol/h·g	none
[23]	Co_3_O_4_	Visible light	42.5 μmol/h·g		ethanol
This work	Co_3_O_4_	Visible light	6.5 μmol/h·g		none
This work	Co_3_O_4_-TiO_2_	Visible light	8.16 μmol/h·g	4.0 μmol/h·g	none
